# Determinants of sexual exposure to HIV in Portuguese and Brazilian adolescents: a path analysis*

**DOI:** 10.1590/1518-8345.6222.3714

**Published:** 2022-09-23

**Authors:** Jules Ramon Brito Teixeira, Shirley Veronica Melo Almeida Lima, Anderson Reis de Sousa, Artur Acelino Francisco Luz Nunes Queiroz, Nilo Manoel Pereira Vieira Barreto, Isabel Amélia Costa Mendes, Inês Fronteira, Álvaro Francisco Lopes de Sousa

**Affiliations:** 1 Universidade Estadual de Feira de Santana, Departamento de Saúde Coletiva, Feira de Santana, BA, Brasil.; 2 Bolsista do Conselho Nacional de Desenvolvimento Científico e Tecnológico (CNPq), Brasil.; 3 Universidade Federal de Sergipe, Departamento de Enfermagem, Lagarto, SE, Brasil.; 4 Universidade Federal da Bahia, Departamento de Enfermagem, Feira de Santana, BA Brasil.; 5 Northwestern University, Institute for Sexual and Gender Minority Health and Well-being, Chicago, IL, Estados Unidos da América.; 6 Universidade de São Paulo, Escola de Enfermagem de Ribeirão Preto, Centro Colaborador da OPAS/OMS para o Desenvolvimento da Pesquisa em Enfermagem, Ribeirão Preto, SP, Brasil.; 7 Universidade Nova de Lisboa, Instituto de Higiene e Medicina Tropical, Lisboa, LX, Portugal.; 8 Centro Universitário UNINOVAFAPI, Teresina, PI, Brasil.

**Keywords:** HIV, Sexual and Gender Minorities, Adolescent, Nursing, Public Health Nursing, Sexual Behavior

## Abstract

**Objective:**

to analyze the direct and indirect effects of determinants of sexual exposure to the human immunodeficiency virus among male adolescents who have sex with men and the implications for nursing care.

**Method:**

cross-sectional study carried out with 578 Portuguese and Brazilian adolescents aged 18 and 19. Interrelationships of conjugal status, use of dating apps, practice of chemsex, unawareness, partner credibility, challenging sexual practices and ineffective forms of protection against sexual exposure to the human immunodeficiency virus were evaluated using the Path Analysis technique.

**Results:**

significant direct effect on sexual exposure to the human immunodeficiency virus: conjugal status (β=-0.16), use of apps (β=-0.30), challenging sexual practices (β=0.48) and ineffective forms of protection (β=0.35). Indirect paths: partner credibility influenced ineffective forms of protection (β=0.77); having a steady/polyamorous relationship influenced the use of dating apps (β=-0.46); chemsex, mediated by challenging sexual practices (β=0.67), determined greater sexual exposure.

**Conclusion:**

adolescent sexual behaviors and forms of amorous/sexual relationship must be considered in nursing care planning to reduce sexual exposure to the human immunodeficiency virus.

## Highlights

(1) Adolescents present high prevalence (81.0%) of sexual exposure to HIV.

(2) Specificities of sexual practices are determinant factors of sexual exposure.

(3) The higher the credibility given to a partner, the higher the sexual exposure to HIV.

(4) Challenging sexual practices (such as fisting) determine sexual exposure.

(5) Nursing care must focus on lowering exposure to HIV.

## Introduction

Adolescents (10 to 19 years), particularly those belonging to key populations such as men who have sex with men (MSM), continue to be disproportionately affected by the human immunodeficiency virus (HIV). In 2016, around 2.1 million people aged 10 to 19 were living with HIV; 260,000 were infected by the virus and there was a 30% increase in official records between 2005 and 2016^1^. Estimates for the year 2019 from the Joint United Nations Program on HIV/AIDS (UNAIDS) indicate that 1.7 million adolescents (1,100,000-2,400,000) were living with HIV worldwide, and two out of seven new infections in that same year occurred in the age group of 15 to 24 (young people)^2^.

The number of adolescent deaths due to HIV/AIDS-related illnesses tripled between 2000 and 2015, the only age group with a consistent increase. In 2016, 55,000 adolescents lost their lives to the virus, the leading cause of death among this group in African countries and the second leading cause among adolescents worldwide. Even more serious is the fact that half of the people aged 15 to 19 living with HIV in the world were born in South Africa, Nigeria, Kenya, India, Mozambique and Tanzania^3^.

There are many factors why adolescents are at a high risk of acquiring HIV. Adolescence and early adulthood are a critical period of development, with significant physical, cognitive and emotional changes. In addition, in this period people experience increasing personal autonomy and responsibility for their own health. The transition from childhood to adulthood is also seen as a time to explore and deal with peer relationships, gender norms, sexuality and economic responsibility^4^ ^-^ ^5^.

Given this context, there are still gaps in the literature addressing adolescents and their relationship with/exposure to HIV. Studies with limited samples and lack of adequate disaggregation for this age group restrict the available evidence, hindering the implementation of new public policies for this group ^(^ ^2^ ^,^ ^6^, as evidenced by the non-inclusion of adolescents in the HIV strategic plans of several countries^7^, including Brazil^8^ and Portugal^9^.

Studies^10^ ^-^ ^11^ indicate that unprotected sex is the most common cause of HIV infection; however, the factors that determine such exposure are poorly understood. Although the literature points to lack of knowledge about HIV and the means of preventing it as the main associated factor, occasional unprotected sex and drug use are also commonly mentioned^12^. Adolescents from key populations are generally less aware of HIV risks or less able to mitigate them compared to their more experienced peers^13^ ^-^ ^14^.

In this context, nursing professionals are strategically positioned and can contribute clinical and behavioral measures for the production of care, both locally and globally, besides health education/literacy with a focus on coping with HIV, with consequent qualification of care and encouragement of teamwork through interprofessional cooperation^15^.

However, there are still many challenges to be faced, which is why this study focuses on a subject matter related to science and nursing practice, which reinforces its rationale, usefulness and social-scientific relevance.

Therefore, the objective was to analyze the direct and indirect effects of determinants of sexual exposure to HIV among male adolescents who have sex with men and the implications for nursing care.

## Method

### Type of study

Cross-sectional study with data from the “You_In-PrEP” project, an international survey carried out throughout Brazil and Portugal with adolescents aged 18 and 19, from January 2020 to May 2021, led by Instituto de Higiene e Medicina Tropical (IHMT) in partnership with the University of São Paulo (USP).

### Population, sample and eligibility criteria

A simple sample calculation for the proportion was performed using G*Power software (version 3.1.9.7), considering the male population aged 18 and 19 in both countries, with a presumed prevalence of 50% (in order to maximize the sample and considering that this is a phenomenon for which there were no prevalence data), a tolerable standard error of 3% and a confidence level of 95%, with the final sample of 578 MSM. There were no lost or discarded survey forms.

### Data collection

The participants were adolescents aged 18 and 19 born in one of the nine countries of the Community of Portuguese Language Countries (CPLP). Participants who were already using Pre-Exposure Prophylaxis (PrEP) and those who were HIV positive were excluded. 

The adolescents who were part of the sample were recruited online through two strategies. The first was snowball sampling, adapted to the virtual environment and consolidated by other studies^16^ ^-^ ^20^. Through this method, the participants themselves are responsible for recruiting other individuals in similar situations through their social and contact networks. Following the method’s criteria, we selected 30 MSM with different characteristics, namely: region or district of residence in each country; race/skin color (white/non-white); income and educational level (primary, secondary or tertiary). These were the first participants, who were called *seeds*.

To identify the seeds, two experienced researchers, properly trained and calibrated, created a public profile on two geolocation-based dating apps (Grindr and Hornet), and via direct chat with online users sent each participant a link to the survey, instructing them to invite other MSM from their social network until the required sample was obtained - dissemination strategy. At this stage, the study approached the first individuals who were available online in each of the two apps and met the inclusion criteria, as recommended by previous studies^14^ ^,^ ^16^ ^-^ ^17^.

Concurrently, as a second recruitment strategy, the researchers also promoted the research on two social media sites, Facebook and Instagram, targeting the MSM population aged 18 and 19. The social media were used as an additional resource due to their ability to reach people outside metropolitan areas, which is absolutely necessary in the case of a continental-sized country like Brazil^18^ ^-^ ^20^. Only individuals who self-identified as men (cisgender or transgender) and aged 18 and 19 were included. Tourists and non-Portuguese speakers were excluded.

In this research, adolescents were considered to be youngsters aged 18 and 19, based on the definitions of the World Health Organization (WHO)^21^, respecting the minimum age criteria for using the investigated apps.

### Data collection instrument

The survey form was hosted on the SurveyMonkey platform for data collection in two versions, with security features that allow only one response per Internet Protocol (IP). Since there are important linguistic differences between the two countries studied, the form was made available in two versions, Brazilian Portuguese and European Portuguese. It was previously validated (face-content validity) by 10 expert judges on the subject, five from each country. There was also a pre-test with five participants from each country.

The form is divided into five sections with 40 questions, most of which are multiple choice. The questions addressed social and demographic information (gender identity, sexual orientation, age, education level, country of residence, country of origin, length of residence in the country); sexual and affective relationships (type of partners, type of relationship, number of partners); knowledge about ways to prevent HIV/AIDS; sexual behaviors and practices; forms of protection used; use of health services; and use, consumption, and willingness to use PrEP.

### Study variables

The study’s dependent variable was recent sexual exposure to HIV among adolescents. Two variables were used for the construction of this indicator: a) sexual position of preference and b) recurrent practice of bareback sex (with response options 0-never, 1-sometimes and 2-always − this variable was dichotomized into 0-no for never and 1-yes for sometimes and always).

The independent variables included in the analyses were:

- Conjugal status: dichotomized into 0-single and 1-in relationship (steady or polyamorous);
- Use of dating apps: 0-no and 1-yes;
- Practice of chemsex: 0-no and 1-yes;
- Unawareness: this indicator was constructed by evaluating agreement with the statements about the main forms of HIV/AIDS prevention - “it is possible to prevent HIV infection by taking a daily pill (PrEP)”; “post-exposure prophylaxis (PEP or PPE) consists of taking pills for a certain period of time (28 days) after unprotected sexual exposure and may stop HIV infection”; and “people living with HIV but whose viral load has not been detected for at least 6 months are not able to transmit the virus to their partners.” The answer options for each statement were 0-yes (aware) and 1-no (unaware); by adding the scores assigned to the three items, an unawareness gradient was constructed ranging from 0 (aware of all information) to 3 (unaware of all information).
- Partner credibility: this indicator was constructed by evaluating four attributes used by adolescents as criteria for having unprotected sex with their sexual partners, namely partner reports taking PrEP; partner is known; partner reports having no sexually transmitted infections (STIs); partner reports having been tested recently. The answers to these items were 0-yes and 1-no. For the construction of the indicator, those who answered no to all items were considered to have a reliable partner (0) − less exposed to the outcome; those who answered yes to at least one item were treated as having an unreliable/poorly reliable partner (1) − exposure category in the analyses.
- Challenging sexual practices: these were evaluated through positive answers to the variables “practices cruising,” “practices double penetration” and “practices fisting.” Adolescents who answered no to the three items were considered 0-no (less exposed) and those who practiced one and/or all three sexual practices were considered 1-yes (exposure category).
- Ineffective forms of protection: the investigated variables used to construct this indicator were those related to the determining reasons for adolescents to engage in unprotected sex, i.e., practicing withdrawal; being the one who penetrates/only top; getting oneself or one’s partners tested before sex; not using any form of prevention. The answer options were 0-does not use this form of protection and 1-uses this form of protection. Those who answered no to all ineffective forms of protection were treated as 0-no (less exposed); those who answered yes to at least one form were treated as 1-yes (exposure category). The variable “ineffective forms of protection” was considered the main exposure for the occurrence of the outcome, that is, sexual exposure to HIV.

### Conceptual framework and study hypotheses

**Figure 1:**
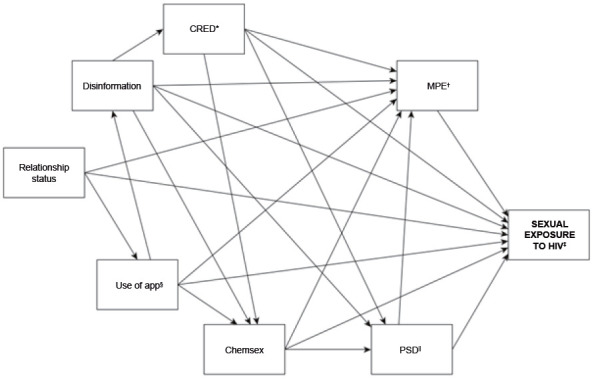
Conceptual framework of determinants of sexual exposure in male adolescents who have sex with men

Considering the conceptual framework of sexual exposure to HIV, a hypothetical model was developed (Figure 1) of the determinants in male adolescents who have sex with men. This conceptual framework, containing the study hypotheses, was represented by means of a Directed Acyclic Graph (DAG)^22^ ^-^ ^23^, in which the variables, treated in this study as directly observed, are represented by rectangles. In this DAG, the relationship between the variables (vertices) is indicated by arrows (edges) that connect them in order to establish causal relationships that are direct (when an arrow goes from one variable to another) or indirect (when there is one or more mediating variables in the causal path between a variable and the outcome)^22^ ^-^ ^23^.

*CRED = Partner credibility; ^†^MPE = Ineffective forms of protection; ^‡^HIV = Human Immunodeficiency Virus; ^§^App = Dating apps; ^||^PSD = Challenging sexual practices

### Data treatment and analysis

The data were initially stored in the Statistical Package for Social Science (SPSS) software database, version 24. First, descriptive analyses of the variables of interest were performed, estimating absolute and relative frequencies, with respective 95% confidence intervals, since all variables were treated as categorical. Next, the database was exported to a format compatible with Mplus software, version 8.7, in which the Path Analysis technique was processed.

Path analysis is an extension of multiple regression, which enables the analysis of complex models and causal relationships. The paths between the variables, whether direct or indirect, are quantitatively expressed through standardized path coefficients (regression coefficients) and aim to determine the strengths of the paths hypothesized in the path diagram^24^. As this is modeling with categorical data, the Weighted Least Squares Means and Variance Adjusted (WLSMV) estimator was used. The Modification Indexes (MI≥10) and the Expected Parameter Changes (EPC≥0.25) were jointly evaluated to identify the need to respecify the modeling paths^25^. Goodness of fit was evaluated using the Root Mean Square Error of Approximation (RMSEA), which must have an index below 0.06 (accepting up to 0.08) with the respective 90% confidence interval below 0.08^25^; Comparative Fit Index (CFI) and Tucker-Lewis Index (TLI) above or equal to 0.95^26^; and Chi-Square normalization (X^2^/degrees of freedom) below 2, as an indication of excellent fit^27^.

### Ethical and legal aspects

The research was approved by the Research Ethics Committee of IHMT, of Universidade Nova de Lisboa, and the Research Ethics Committee of the Ribeirão Preto School of Nursing, of the University of São Paulo (USP). The ethical standards in force in both countries were met, respecting the age criteria of the apps, and a free and informed consent form was provided online for the participants’ consent. At the end of the research they had access to institutional websites to obtain information on HIV/AIDS prevention.

## Results

A total of 578 adolescents took part in the study, with a predominance of 18 years of age (52.4%), male sex assigned at birth (96.5%), Brazilian (63.5%), residing in Brazil (50.3%), single (69.4%), with a casual/occasional sexual partner (69.7%), having had one to two sexual partners in the last 30 days (53.5%) and with a preference for versatile sexual position (39.1%). Among those who were in a relationship (Table 1), most were non-practitioners of chemsex (69.2%), users of dating apps (82.0%) and had sexual partners found through apps (81.3%). Great preference for the receptive or bottom position in sexual intercourse (506; 87.5%; 95%CI=62.6-70.4) and a high frequency of bareback (unprotected anal sex) practitioners (339; 58.7%; 95%CI=54.5-62.7) were observed, indicating that the majority showed sexual exposure to HIV (468; 81.0%; 95%CI=77.5-84.1), with a slightly higher proportion in Portugal (81.2%; 95%CI=76.2-85.5) compared to Brazil (80.8%;95%CI=75.7-85.1).

**Table 1:** Sociodemographic and sexual characteristics of male adolescents who have sex with men (n=578). Brazil, Portugal, 2021

Variables	n	%	*95%CI
**Age**			
18	303	52.4	48.3-56.6
19	275	47.6	43.4-51.7
**Sex assigned at birth** (n=577)			
Male	557	96.5	94.7-97.9
Female	20	3.5	2.1-5.3
**Education**			
Primary	8	1.4	0.6-2.7
Secondary	217	37.5	33.6-41.6
Attending tertiary	353	61.1	57.0-65.1
**Country of birth**			
Brazil	367	63.5	59.4-67.4
Portugal	202	34.9	31.1-39.0
Mozambique	4	0.7	0.2-1.8
Guinea Bissau	4	0.7	0.2-1.8
Cape Verde	1	0.2	0.004-0.10
**Country of residence**			
Brazil	291	50.3	46.2-54.5
Portugal	287	49.7	45.5-53.8
**Relationship status**			
Single	401	69.4	65.4-73.1
Steady relationship	149	25.8	22.2-29.5
Polyamorous relationship	28	4.8	3.2-6.9
**Sexual partner**			
Casual/occasional partner	403	69.7	65.8-73.4
Steady/usual partner	28	4.8	3.2-6.9
Steady partner and occasional partners	147	25.4	21.9-29.2
**Number of partners in the last 30 days**			
None	97	16.8	13.8-20.1
1 to 2	309	53.5	49.3-57.6
3 or more	172	29.8	26.1-33.7
**Sexual position of preference**			
Oral sex only	21	3.6	2.26-5.50
Penetrating/top - exclusively	51	8.8	66-11.4
Penetrating/top - mostly	101	17.5	14.5-20.8
Being penetrated/bottom - exclusively	68	11.8	9.3-14.7
Being penetrated/bottom - mostly	111	19.2	16.1-22.7
Penetrating and being penetrated (versatile) alike	226	39.1	35.1-43.2
**Chemsex practice**			
No	400	69.2	65.3-72.9
Yes	178	30.8	27.1-34.7
**Use of apps^†^** ^†^			
No	104	18.0	14.9-21.4
Yes	474	82.0	78.6-85.1
**Sexual partners found through apps**			
No	108	18.7	15.6-22.1
Yes	470	81.3	77.9-84.4

*95%CI = 95% Confidence Interval; ^†^Apps = Dating apps

As for the variables that made up the unawareness indicator, a higher number of adolescents did not know that PEP, taken after sexual exposure, can interrupt HIV infection (32.9%). Regarding partner credibility, among the reasons given for having unprotected sex, most chose to do so when the partner was known/repeated (26.8%). Among challenging sexual practices, double penetration predominated (26.8%), and regarding ineffective forms of protection, there was a greater number of adolescents who reported practicing withdrawal (26.8%) (Table 2).

**Table 2:** Breakdown of male adolescents who have sex with men (n=578) by indicators of unawareness, partner credibility, challenging sexual practices and ineffective forms of protection. Brazil, Portugal, 2021

Variables	n	%	*95%CI
**Unawareness (is unaware)**			
Is it possible to prevent HIV^†^ infection by taking PrEP^‡^ daily	66	11.4	8.9-14.3
PEP^§^ taken after sexual exposure can stop HIV^†^ infection	190	32.9	29.1-36.9
People with HIV^†^ with undetected viral load for at least 6 months do not transmit the virus	140	24.2	20.8-27.9
**Partner credibility**			
Partner using PrEP^‡^	57	9.9	7.6-12.6
Known/repeated partner	155	26.8	23.2-30.6
Partner claims not to have STD^||^	60	10.4	8.0-13.2
Partner claims being recently tested	41	7.1	5.1-9.5
**Challenging sexual practices**			
Cruising	23	4.0	2.5-5.9
Double penetration	155	26.8	23.2-30.6
Fisting	60	10.4	8.0-13.2
**Ineffective forms of protection**			
Coitus interruptus	155	26.8	23.2-30.6
Only penetrates	32	5.5	3.8-7.7
Does not use any form of prevention	60	10.4	8.0-13.2
Tests self or partners before sex	127	22.0	18.7-25.6
Keeps viral load low	34	5.9	4.1-8.1

^*^95%CI = 95% Confidence Interval; ^†^HIV = Human Immunodeficiency Virus; ^‡^PrEP = Pre-Exposure Prophylaxis; ^§^PEP = Post-Exposure Prophylaxis; ^||^STD = Sexually Transmitted Diseases

The path analysis modelling of determinants of sexual exposure to HIV in male adolescents who have sex with men revealed adequate goodness of fit: X2/gl=1.4; RMSEA=0.027 (90%CI=0.000-0.065); CFI=0.997 and TLI=0.985.

**Figure 2:**
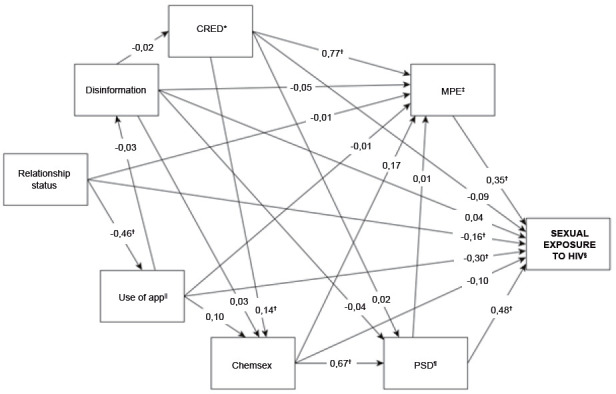
Conceptual framework of determinants of sexual exposure in male adolescents who have sex with men

The following variables showed a statistically significant direct effect on sexual exposure among male adolescents who have sex with men: challenging sexual practices (β=0.48; p-value=0.001), ineffective forms of protection (β=0.35; p- value=0.038), use of dating apps (β=-0.30; p-value=0.004) and conjugal status (β=-0.16; p-value<0.046). It was identified that respondents with higher rates of challenging sexual practices and ineffective forms of protection, who do not use dating apps or who had a steady partner or polyamorous relationship, showed a higher level of sexual exposure. We observed a high direct effect of challenging sexual practices, moderate use of apps and ineffective forms of protection, and a small effect of conjugal status, showing that challenging sexual practices was the factor that most contributed to sexual exposure (Figure 2).

*CRED = Partner credibility; ^†^Statistically significant (p<0.05); ^‡^MPE = Ineffective forms of protection; ^§^HIV = Human Immunodeficiency Virus; ^||^App = Dating apps; ^¶^PSD = Sexually challenging practices

In the evaluation of the statistically significant specific indirect paths, it was identified that: a) the greater the partner credibility, the greater the rate of ineffective forms of protection and, consequently, the greater the sexual exposure, with a strong effect of partner credibility on ineffective forms of protection (β=0.77; p-value<0.001); b) having a steady partner or polyamorous relationship was associated with a higher level of dating app use (β=-0.46; p-value<0.001) and lower sexual exposure; c) there was no significant direct effect of chemsex on sexual exposure (β=-0.10; p-value=0.528); however, when mediated by challenging sexual practices, it was found that the higher the frequency of chemsex, the greater the rate of challenging sexual practices (β=0.67; p-value<0.001) and sexual exposure; d) the greater the partner credibility, the greater the frequency of chemsex (β=0.14; p-value=0.037), challenging sexual practices and sexual exposure, with a strong effect of chemsex on challenging sexual practices; e) the level of challenging sexual practices was an important mediator of the relationship between dating app use, partner credibility and chemsex with sexual exposure among adolescents (Figure 2).

The largest total effects on sexual exposure of male adolescents who have sex with men were observed for higher rates of challenging sexual practices (β=0.481; p-value<0.001) and ineffective forms of protection (β=0.347; p-value=0.038). The effect of challenging sexual practices was strong and that of ineffective forms of protection was moderate - both significant. There were also average and significant total effects of practicing chemsex (β=0.285; p-value<0.001), not using dating apps (β=-0.273; p-value=0.011) and greater partner credibility (β=0.222; p-value<0.001) (Table 3).

**Table 3:** Standardized total and indirect effects of the path analysis modelling of determinants of sexual exposure in male adolescents who have sex with men (n=578). Brazil, Portugal, 2021

Paths	Standardized coefficient	p-value
**Total effects**		
APP*→EXP^†^	-0.273	0.011
CHEM^‡^→EXP^†^	0.285	<0.001
DES^§^→EXP^†^	0.049	0.449
PSD^||^→EXP^†^	0.481	0.001
MPE^¶^→EXP^†^	0.347	0.038
CRED**→EXP^†^	0.222	0.003
CONJ^††^→EXP^†^	-0.038	0.536
**Specific indirect effects**		
APP*		
APP*→CHEM^‡^→EXP^†^	-0.009	0.553
APP*→DES^§^→EXP^†^	-0.001	0.696
APP*→MPE^¶^→EXP^†^	-0.001	0.972
APP*→DES^§^→CHEM^‡^→EXP^†^	0.000	0.790
APP*→CHEM^‡^→PSD^||^→EXP^†^	0.031	0.225
APP*→DES^§^→PSD^||^→EXP^†^	-0.001	0.699
APP*→CHEM^‡^→MPE^¶^→EXP^†^	0.006	0.391
APP*→DES^§^→MPE^¶^→EXP^†^	0.000	0.693
APP*→DES^§^→CRED**→EXP^†^	0.000	0.828
APP*→DES^§^→CRED**→CHEM^‡^→EXP^†^	0.000	0.832
APP*→DES^§^→CHEM^‡^→PSD^||^→EXP^†^	0.000	0.770
APP*→DES^§^→CRED**→PSD^||^→EXP^†^	0.000	0.871
APP*→DES^§^→CHEM^‡^→MPE^¶^→EXP^†^	0.000	0.776
APP*→CHEM^‡^→PSD^||^→MPE^¶^→EXP^†^	0.000	0.891
APP*→DES^§^→PSD^||^→MPE^¶^→EXP^†^	0.000	0.900
APP*→DES^§^→CRED**→MPE^¶^→EXP^†^	0.000	0.820
APP*→DES^§^→CRED**→CHEM^‡^→PSD^||^→EXP^†^	0.000	0.821
APP*→DES^§^→CRED**→CHEM^‡^→MPE→EXP^†^	0.000	0.823
APP*→DES^§^→CHEM^‡^→PSD^||^→MPE^¶^→EXP^†^	0.000	0.901
APP*→DES^§^→CRED**→PSD^||^→MPE^¶^→EXP^†^	0.000	0.906
APP*→DES^§^→CRED**→CHEM^‡^→PSD^||^→MPE^¶^→EXP^†^	0.000	0.909
**CONJ** ^††^		
CONJ^††^→APP*→EXP^†^	0.136	0.010
CONJ^††^→MPE^¶^→EXP^†^	-0.003	0.888
CONJ^††^→APP*→CHEM^‡^→EXP^†^	0.004	0.552
CONJ^††^→APP*→DES^§^→EXP^†^	0.001	0.698
CONJ^††^→APP*→MPE^¶^→EXP^†^	0.000	0.972
CONJ^††^→APP*→DES^§^→CHEM^‡^→EXP^†^	0.000	0.790
CONJ^††^→APP*→CHEM^‡^→PSD^||^→EXP^†^	-0.014	0.225
CONJ^††^→APP*→DES^§^→PSD^||^→EXP^†^	0.000	0.700
CONJ^††^→APP*→CHEM^‡^→MPE^¶^→EXP^†^	-0.003	0.389
CONJ^††^→APP*→DES^§^→MPE^¶^→EXP^†^	0.000	0.693
CONJ^††^→APP*→DES^§^→CRED**→EXP^†^	0.000	0.829
CONJ^††^→APP*→DES^§^→CRED**→CHEM^‡^→EXP^†^	0.000	0.832
CONJ^††^→APP*→DES^§^→CHEM^‡^→PSD^||^→EXP^†^	0.000	0.771
CONJ^††^→APP*→DES^§^→CRED**→PSD^||^→EXP^†^	0.000	0.871
CONJ^††^→APP*→DES^§^→CHEM^‡^→MPE^¶^→EXP^†^	0.000	0.776
CONJ^††^→APP*→CHEM^‡^→PSD^||^→MPE^¶^→EXP^†^	0.000	0.891
CONJ^††^→APP*→DES^§^→PSD^||^→MPE^¶^→EXP^†^	0.000	0.900
CONJ^††^→APP*→DES^§^→CRED**→MPE^¶^→EXP^†^	0.000	0.821
CONJ^††^→APP*→DES^§^→CRED**→CHEM^‡^→PSD^||^→EXP^†^	0.000	0.821
CONJ^††^→APP*→DES^§^→CRED**→CHEM^‡^→MPE^¶^→EXP^†^	0.000	0.824
CONJ^††^→APP*→DES^§^→CHEM^‡^→PSD^||^→MPE^¶^→EXP^†^	0.000	0.901
CONJ^††^→APP*→DES^§^→CRED**→PSD^||^→MPE^¶^→EXP^†^	0.000	0.907
CONJ^††^→APP*→DES^§^→CRED**→CHEM^‡^→PSD^||^→MPE^¶^→EXP^†^	0.000	0.909
**CHEM** ^‡^		
CHEM^‡^→PSD^||^→EXP^†^	0.321	0.002
CHEM^‡^→MPE^¶^→EXP^†^	0.058	0.244
CHEM^‡^→PSD^||^→MPE^¶^→EXP^†^	0.003	0.891
**DES** ^§^		
DES^§^→CHEM^‡^→EXP^†^	-0.002	0.736
DES^§^→PSD^||^→EXP^†^	0.020	0.473
DES^§^→MPE^¶^→EXP^†^	-0.017	0.410
DES^§^→CRED**→EXP^†^	0.001	0.815
DES^§^→CRED**→CHEM^‡^→EXP^†^	0.000	0.819
DES^§^→CHEM^‡^→PSD^||^→EXP^†^	0.008	0.691
DES^§^→CRED**→PSD^||^→EXP^†^	0.000	0.866
DES^§^→CHEM^‡^→MPE^¶^→EXP^†^	0.001	0.706
DES^§^→PSD^||^→MPE^¶^→EXP^†^	0.000	0.894
DES^§^→CRED**→MPE^¶^→EXP^†^	-0.004	0.804
DES^§^→CRED**→CHEM^‡^→PSD^||^→EXP^†^	-0.001	0.804
DES^§^→CRED**→CHEM^‡^→MPE^¶^→EXP^†^	0.000	0.808
DES^§^→CHEM^‡^→PSD^||^→MPE^¶^→EXP^†^	0.000	0.894
DES^§^→CRED**→PSD^||^→MPE^¶^→EXP^†^	0.000	0.903
DES^§^→CRED**→CHEM^‡^→PSD^||^→MPE^¶^→EXP^†^	0.000	0.905
CRED**		
CRED**→CHEM^‡^→EXP^†^	-0.014	0.551
CRED**→PSD^||^→EXP^†^	0.007	0.813
CRED**→MPE^¶^→EXP^†^	0.268	0.040
CRED**→CHEM^‡^→PSD^||^→EXP^†^	0.046	0.094
CRED**→CHEM^‡^→MPE^¶^→EXP^†^	0.008	0.259
CRED**→PSD^||^→MPE^¶^→EXP^†^	0.000	0.887
CRED**→CHEM^‡^→PSD^||^→MPE^¶^→EXP^†^	0.000	0.891
**PSD** ^||^		
PSD^||^→MPE^¶^→EXP^†^	0.005	0.891

^*^APP = Dating app use; ^†^EXP = Sexual exposure to HIV; ^‡^CHEM = Chemsex; ^§^DES = Unawareness; ^||^PSD = Challenging sexual practices; ^¶^MPE = Ineffective forms of protection; **CRED = Partner credibility; ^††^CONJ = Conjugal status

Considering specific indirect paths, there was a small and significant effect of being single on sexual exposure, mediated by the use of dating apps (β=0.136; p-value=0.010). Chemsex, mediated by challenging sexual practices (β=0.321; p-value=0.002), had a moderate effect on sexual exposure among adolescents. Partner credibility, mediated by ineffective forms of protection (β=0.268; p-value=0.040), was associated with greater sexual exposure (Table 3).

## Discussion

In this study, we investigated factors that may determine sexual exposure to HIV in a sample of adolescent MSM, residing in two Portuguese-speaking countries that share a high flow of people in migratory processes. There was high sexual exposure to HIV (81.0%; 95%CI=77.5-84.1), both in Portugal (81.2%; 95%CI=76.2-85.5) and in Brazil (80.8%; 95%CI=75.7-85.1), when comparing the general population of MSM (28.7%)^28^. The findings contrast with the global situation and with the increase in HIV prevention policies in both countries, which have accumulated a significant increase of new inputs that add to a number of classic/traditional strategies dedicated to helping to prevent possible risk of exposure to HIV^14^ ^,^ ^18^ ^,^ ^29^.

However, this finding can be explained by the fact that mere access to knowledge about HIV and forms of prevention may not be sufficient and/or necessarily imply a change in unprotected sexual practices, since they are influenced by both individual and structural aspects, in which stand out, historically, social inequalities, discrimination and stigma, and relational, cultural and subjective aspects that are associated with vulnerability to the HIV virus. In this sense, the findings of our study call upon nursing teams to globally analyze the experiences of adolescents in relation to the context of HIV/AIDS.

The scientific literature has made advances in knowledge about the difficulties of adolescents adhering to HIV protection measures, especially regarding the use of condoms^30^ ^-^ ^32^, which should draw the attention of nursing professionals who work in adolescent health programs as well as those that address health education. However, the factors that can more accurately explain the sexual health behaviors adopted by this key population are still poorly understood, which evidences the pioneering initiative of this study in identifying the determinants for involvement in sexual exposure to HIV in Brazilian and Portuguese adolescent MSM, which are still related to strategic contexts for public health, such as immigration^33^. Analyzing the global contexts of adolescent health may contribute in a comprehensive manner to overcoming disadvantages between different countries, enhancing the dissemination of strategies and good practices and facilitating the socialization of knowledge.

Although they are countries with striking social, cultural and political differences, previous studies^18^ ^-^ ^20^ ^,^ ^28^ have shown that Brazilian and Portuguese MSM present similar sexual behaviors and practices that deserve more in-depth analysis, especially from the perspective of global health. Thus, this study conveys in a convergent manner the need for knowledge translation in order to guide professional conduct in health and nursing for adolescents residing in both countries.

Our analyses showed a direct effect of factors related to the establishment of partnerships (not using dating apps and having a steady partner or polyamorous relationship) and sexual practices (performing challenging sexual practices and adopting ineffective forms of protection) on sexual exposure to HIV. Understanding and recognizing that a considerable number of adolescents around the world are sexually active is an important point in the study of the sexual exposure of adolescents to HIV/STI, and to a certain extent prompts different social actors (managers, public and community leaders, political representatives and healthcare professionals) to engage in the defense of a priority public health agenda for the adolescent population. Studies in several countries indicate that the number of adolescents in a steady relationship exceeds the proportion of those that are single^34^ ^-^ ^36^, but all of them are likely to be involved in casual sexual encounters. In addition, it is important to note that single adolescents tend to have more sexual partners than people over 20 years of age^35^ ^-^ ^37^.

The specifics of establishing relationships and partners seem to be key to understanding the determinants of HIV exposure in adolescents. Having some degree of involvement with the sexual partner, regardless of their HIV status, has led MSM to have unprotected sex^14^ ^,^ ^38^ ^-^ ^40^. Our findings show that the greater the partner credibility, the greater the rate of ineffective forms of protection, the greater the frequency of chemsex and challenging sexual practices and, consequently, the greater the sexual exposure to HIV among adolescents. This behavior is already partly reported in the literature^41^ ^-^ ^42^ and can be explained by a mistaken notion of safety afforded by a stable or even repeated and known partnership.

The distorted view can be supported by emotional rather than factual aspects (such as relying on regular testing instead of condom use), associated with ineffective forms of protection, which contributes to the increase in new cases of STIs among adolescent MSM. As trust and familiarity with the partner are established and the relationship is consolidated, there is a perception that the risk is lower for those involved, who tend to reduce or dispense with the use of condoms when having sex with a partner^37^ ^,^ ^41^ ^,^ ^43^ ^-^ ^44^. Moreover, they may have little knowledge and poor discernment about the efficacy and effectiveness of prevention methods, assuming a high risk of sexual exposure to HIV^13^ ^,^ ^45^, as corroborated in this study.

The risk of acquiring or transmitting HIV varies according to the type and frequency of exposure or established sexual practice. The National Youth Risk Behavior Survey of the Centers for Disease Control and Prevention (CDC), of the United States Department of Health and Human Services, contributes evidence of the main causes of death and disability among adolescents in the United States, which relate to use of illicit substances before sex, low rates of condom use and multiple sexual partners^46^. We identified that the frequency of chemsex, mediated by challenging sexual practices, has a moderate effect on sexual exposure among adolescents. In addition, frequency of challenging sexual practices was an important mediator of the relationship of use of dating apps and partner credibility with sexual exposure among adolescents.

HIV prevention in countries with relatively stable prevalence or where the epidemic is concentrated in specific populations, such as Brazil and Portugal, is especially challenging, as the spread of the virus is fueled by high-risk and often stigmatized behaviors^47^ ^-^ ^48^.

So-called unconventional sexual practices are rooted in different aspects of sexuality and socialization, and some of them may be connected with chemsex. These factors present an inherent dualism (pain and pleasure, sobriety and disinhibition) that is not necessarily exclusionary and may coexist during sex, albeit in a conflicting way^49^. The search for pushing sexual boundaries, whether literally or figuratively, developed within homosexual identities and practices in a queer perspective, shows that such practices are reflections of the non-belonging of sex between men in society, thus being execrated in its concept^50^.

Adolescence is understood as the typically healthy period of life; however, the HIV/STI diagnosis makes this phase more complex and challenging, considering the insufficient psychosocial maturity of teens to deal “alone” with the onset of diseases and conditions, especially infectious and chronic ones such as HIV^51^ ^-^ ^53^.

That said, it is worth reflecting on the defense of the priority agenda for adolescent health in order to assume a public, social and citizenship commitment to the protection, safety, autonomy, empowerment and self-management of adolescents, especially in countries that speak the same language, which can configure a favorable setting for action in public health. A strong and efficient primary healthcare system is capable of addressing this context, proposing and implementing biomedical and behavioral measures to cope with HIV/AIDS that qualify care and encourage teamwork with interprofessional cooperation, providing comprehensive healthcare for adolescents.

Based on the findings about the dynamics of sexual practices in adolescence, it is possible to clarify some implications for nursing care and practice, which can better direct therapeutic behaviors, literacy and guidance in health, expansion of testing coverage for HIV and other STIs, and access and adherence to PrEP and PEP services and therapies and health programs aimed at this population. In addition, the findings can help implement care guidelines and protocols and direct individual and collective interventions used by nursing teams with adolescents in their respective countries.

It is beneficial to create a space for nursing care that is conducive to listening and dialogue and free of judgment, with positive effects on the establishment of bonds, confidentiality and accountability between professionals and individuals/users. Advances in nursing and healthcare practices aimed at adolescent MSM are urgent, given the rapid transformations in the construction of social and sexual identities, in sociability (analog and virtual), in the early and frequent consumption of pornographic content, alcohol and other drugs in sex and in early sexual initiation.

In this sense, nursing care plans need to incorporate coherent, contextualized, inclusive and accurate interventions based on evidence, plus an intersectional approach aimed at capturing the influences, overlaps and erasure generated by social markers of difference and/or structural and structuring categories, namely: gender/gender identity, sexuality/sexual orientation, race/ethnicity, class/economic situation, age/generation, country/geographical location and others that are essential in the context of production of care.

There is a need for a diagnostic proposition of results and interventions grounded in nursing references - theoretical models, nursing theories - as an exercise to strengthen specific nursing knowledge. Thus, the findings of our study may contribute to the production of unique care aimed at the adolescent population, with the structuring of nursing care guidelines and the creation of community health promotion strategies in interface with focal public policies, such as the National Policy for Comprehensive Adolescent Health Care, in force in Brazil, and health programs at Ibero-American level.

It is also hoped that such initiatives will encourage nurses and nursing teams to interact with other health categories and related areas and produce care adapted to sociocultural realities in an intersectional approach, considering the nuances and specificities of adolescence in Brazil and Portugal, as in other countries, aiming to establish cooperative interprofessional practice in which adolescent MSM and their social determinants are at the core of health care.

This survey has some limitations that must be considered. The observational design of the study, with the use of path analysis, made it possible to identify possible causal relationships between the independent variables and the outcome of sexual exposure to HIV among adolescents, but not to establish causal relationships between those factors. The use of an online form limited the sample to adolescents who could afford to have access to smartphone/computer and internet connection. The characteristics of the dating apps made it impossible for adolescents under the age of 18 to take part in the study.

## Conclusion

In our sample, Brazilian and Portuguese adolescent MSM had a high prevalence of sexual exposure to HIV. Among the various possible causal relationships, the sexual behaviors and practices of adolescents as well as the forms of amorous/sexual relationship were determining for exposure and must be considered in nursing care planning aimed at this population in order to reduce exposure to the virus. Nursing leadership in healthcare teams must be committed to taking this crucial issue to decision-making bodies in charge of defining national health policies for the comprehensive healthcare of adolescents, so that individual or collective, protective and preventive measures are explicitly defined and adopted in the national health services of the countries and thus implemented by professionals prepared to perform their due role.
